# Training and Practices of Cannabis Dispensary Staff

**DOI:** 10.1089/can.2016.0024

**Published:** 2016-12-01

**Authors:** Nancy A. Haug, Dustin Kieschnick, James E. Sottile, Kimberly A. Babson, Ryan Vandrey, Marcel O. Bonn-Miller

**Affiliations:** ^1^ PGSP-Stanford Psy.D. Consortium, Palo Alto University, Palo Alto, California.; ^2^ Department of Psychiatry and Behavioral Sciences, Stanford University School of Medicine, Stanford, California.; ^3^ Pacific Graduate School of Psychology, Palo Alto University, Palo Alto, California.; ^4^ National Center for PTSD, VA Palo Alto Healthcare System, Palo Alto, California.; ^5^ Department of Psychiatry and Behavioral Sciences, Johns Hopkins University School of Medicine, Baltimore, Maryland.; ^6^ Center for Innovation to Implementation, VA Palo Alto Healthcare System, Palo Alto, California.; ^7^ Center of Excellence in Substance Abuse Treatment and Education, Philadelphia VAMC, Philadelphia, Pennsylvania.; ^8^ Department of Psychiatry, University of Pennsylvania Perelman School of Medicine, Philadelphia, Pennsylvania.

**Keywords:** cannabis, marijuana, dispensary, practices

## Abstract

**Introduction:** The proliferation of cannabis dispensaries within the United States has emerged from patient demand for the legalization of cannabis as an alternative treatment for a number of conditions and symptoms. Unfortunately, nothing is known about the practices of dispensary staff with respect to recommendation of cannabis strains/concentrations for specific patient ailments. To address this limitation, the present study assessed the training and practices of cannabis dispensary staff.

**Materials and Methods:** Medical and nonmedical dispensary staff (*n*=55) were recruited *via* e-mail and social media to complete an online survey assessing their demographic characteristics, dispensary features, patient characteristics, formal training, and cannabis recommendation practices.

**Results:** Fifty-five percent of dispensary staff reported some formal training for their position, with 20% reporting medical/scientific training. A majority (94%) indicated that they provide specific cannabis advice to patients. In terms of strains, dispensary staff trended toward recommendations of Indica for anxiety, chronic pain, insomnia, nightmares, and Tourette's syndrome. They were more likely to recommend Indica and hybrid plants for post-traumatic stress disorder (PTSD)/trauma and muscle spasms. In contrast, staff were less likely to recommend Indica for depression; hybrid strains were most often recommended for amyotrophic lateral sclerosis (ALS). In terms of cannabinoid concentrations, dispensary staff were most likely to recommend a 1:1 ratio of delta-9-tetrahydrocannabinol (THC):cannabidiol (CBD) for patients suffering from anxiety, Crohn's disease, hepatitis C, and PTSD/trauma, while patients seeking appetite stimulation were most likely to be recommended THC. Staff recommended high CBD for arthritis and Alzheimer's disease and a high CBD or 1:1 ratio for ALS, epilepsy, and muscle spasms.

**Conclusions:** Although many dispensary staff are making recommendations consistent with current evidence, some are recommending cannabis that has either not been shown effective for, or could exacerbate, a patient's condition. Findings underscore the importance of consistent, evidence-based, training of dispensary staff who provide specific recommendations for patient medical conditions.

## Introduction

Approximately half of the United States has legalized cannabis for medicinal purposes, with four states having also legalized cannabis for nonmedical use. Epidemiological research suggests that despite its association with negative short- and long-term effects such as addiction, deficits in cognitive performance and motor coordination, and psychosis,^1^ a number of particularly vulnerable groups of individuals are using cannabis to alleviate their medical conditions (e.g., anxiety, chronic pain, epilepsy, cancer, HIV/AIDS, post-traumatic stress disorder [PTSD]).^2,3^ Indeed, more than 1,000 medical cannabis dispensaries, cooperatives, and delivery services are operating in California,^4^ and ∼500 exist in Colorado, to meet patient demand.

Although each state has created its own legislation to govern the cultivation and distribution of cannabis to individuals, there is currently little to no guidance or oversight of associated patient care. Indeed, with the exception of a few states that have mandated cannabis-specific physician continuing medical education (e.g., New York), the majority of states do not require any training for either those providing “recommendations” for patient cannabis use (i.e., physicians) or those actually dispensing cannabis to consumers (i.e., dispensaries and/or “bud tenders”). This is troubling, as cannabis comprises more than 400 chemical compounds and is associated with widely variable effects among humans.^5^ To provide a specific example, empirical literature has shown that delta-9-tetrahydrocannabinol (THC), the primary psychoactive compound in the cannabis plant, can be anxiogenic, while cannabidiol (CBD), a secondary cannabinoid, has anxiolytic effects.^6^ Next, the literature would suggest that the provision of cannabis, comprising high levels of THC, to individuals with anxiety may be contraindicated.

Although it is important to note that rigorous research on the use of cannabis as a therapeutic remains in its relative infancy, issues of inconsistent and nonempirically supported practices by physicians plague the cannabis and substance use field more broadly.^7^ So, to offer initial information regarding current practices by those providing cannabis recommendations to patients, the present study aimed to document the training and practices of a sample of dispensary staff (i.e., “bud tenders”). Given its descriptive nature, no specific hypotheses were forwarded; however, information garnered from this study is meant to inform targeted implementation science efforts aimed at streamlining provider practices and highlighting the need for structured education in this emerging industry that has developed outside traditional mechanisms of medical drug development.

## Materials and Methods

### Participants

Participants included 55 self-identified dispensary staff members who provided informed consent to complete an online survey. Dispensary types included medical (59%), nonmedical (18%), and both medical and nonmedical (23%). The dispensaries were located in Colorado (41%), California (20%), Arizona (16%), Oregon (2%), District of Columbia (5%), and the Northeast (10%; Connecticut, Rhode Island, Massachusetts, Maine). The locations of the dispensaries were self-reported as follows: rural (16%), suburban (13%), small city (<300,000; 35%), and large city (>300,000; 36%).

### Measures

An online survey was constructed by the study investigators to evaluate the training, knowledge, attitudes, and practices of dispensary staff. Questions included demographics (i.e., age, race/ethnicity, sexual orientation, marital status, education, annual income, dispensary earnings, and hours worked), dispensary features (i.e., geographical location, zip code, type of dispensary), and a checklist of primary responsibilities. A dichotomous (yes/no) item assessed formal dispensary training, and if endorsed, the item branched to a checklist of types of training (e.g., medical, scientific, business, customer service) with a textbox for “other.” Additional items assessed the number of patients served, the percentage of patients who are repeat patrons, and a categorical response choice for how often repeat customers visit the dispensary. The typical amount of cannabis purchased per visit was assessed by having the participant fill in an amount in grams, ounces, or dollars. A dichotomous (yes/no) item assessed whether advice, guidance, or counsel is provided to patients, and if endorsed, the item branched to a checklist of types of advice (e.g., benefits of cannabis and side effects) with a textbox for “other.” Items assessing the medical/psychological symptoms or conditions reported by patients were indexed on a 3-point Likert scale ranging from “rarely” to “frequently.” Respondents also checked off which cannabinoid concentrations (i.e., high THC, high CBD, 1:1 ratio of THC/CBD) or cannabis plant strain recommendations (i.e., sativa, indica, hybrid) they made for each of the symptoms or conditions listed. Attitudes toward work (e.g., satisfaction, feeling valued, stigma, burnout) were assessed on a 5-point Likert scale ranging from “very satisfied” to “very dissatisfied” or “always” to “never.”

### Procedure

The study was approved by the Palo Alto University Institutional Review Board (FWA00010885; Protocol# 15-001-S). A Federal Certificate of Confidentiality was obtained from the National Institutes of Health to protect participant confidentiality. Dispensaries were identified *via* website finders (i.e., leafly.com, weedmaps.com) and a contact list from the Americans for Safe Access. From September 2015 through May 2016, dispensary staff were invited *via* e-mail (*n*=550, with 10% returned as undeliverable and 20% providing an automated e-mail response) and/or telephone (*n*=117) to complete an anonymous survey. A direct link to the survey was also posted on a cannabis advocacy organization Facebook page (i.e., National Organization for the Reform of Marijuana Laws) and a Reddit subreddit geared toward dispensary staff. Of those who accessed the survey or clicked on the survey link, 87% provided informed consent to continue. On completion of the survey, 33 participants were placed in a lottery drawing for a $25 Amazon.com gift card. To increase rates of participation in a later wave of recruitment, each participant was compensated with a $10 Amazon.com gift card (*n*=22).

Survey data were analyzed cross-sectionally using IBM SPSS Statistics, version 23. Descriptive statistics provided a profile of dispensary staff characteristics. One-sample *t*-tests for proportions were calculated to compare cannabinoid and strain recommendations for each patient symptom or condition. Since the survey allowed for participants to skip items, the reported numbers for each item may differ.

## Results

### Participant characteristics

The mean age of participants was 31.9 years (standard deviation [SD]=9.8 years), with a range of 22–63 years. Majority were Caucasian (86%) and 9% identified as having Hispanic or Latino ethnicity. The sexual orientation of participants was heterosexual (67%), bisexual (22%), homosexual (7%), and asexual (4%). The sample was 55% female, 33% reported being married or partnered, and 60% had a college degree or higher. Most (84%) reported working at the dispensary full-time (>30 h) at an average of $15.00/h (SD=$4.60; Range=$8.00–$25.00/h). The average duration of employment at the current dispensary ranged from 1 month to 7 years with a median of 1 year (M=21.6 months, SD=20.0 months). Twenty percent of the sample reported working at another dispensary before their current position.

### Dispensary staff training and responsibilities

Dispensary staff were asked whether they received any formal training for their current position and the type of training received. In our sample, 55% (*n*=30) of staff members reported some formal training. The types of training included customer service (35%; *n*=19), business (26%; *n*=14), medical (20%; *n*=11), other (20%; *n*=11), and scientific (13%; *n*=7). Other training consisted of “bud tender” certification, or courses on cannabis (e.g., Cannabis 101), or safety and regulatory compliance (e.g., SellSmart, METRC).

The dispensary staff described their primary job responsibilities as follows: customer service (91%; *n*=39), stocking inventory (79%; *n*=34), ordering supplies or dealing with vendors/growers (67%; *n*=29), counseling patients (63%; *n*=27), record-keeping (63%; *n*=27), budgeting/finances/accounting (46%; *n*=20), and other responsibilities (25%; *n*=14) such as human resources, delivery, marketing, packaging products, and creating signage.

### Dispensary patients

The number of patients served by the dispensaries ranged from 15 to 5,000 patients per week, with a median of 425 patients (M=778.4, SD=1001.5, *n*=50). On average, M=69% (SD=21%) of patients were described as repeat or frequent patrons. Repeat patrons visited the dispensary daily (26%; *n*=13), 2 to 3 times per week (40%; *n*=20), once a week (22%; *n*=11), or 2 to 3 times per month (12%; *n*=6). The average amount of cannabis purchased per visit was reported as M=10.4 g (SD=9.4 g, *n*=31) or M=$83.00 (SD=$32.00, Range=$25.00–$150.00, *n*=18).

**FIG. 1.**
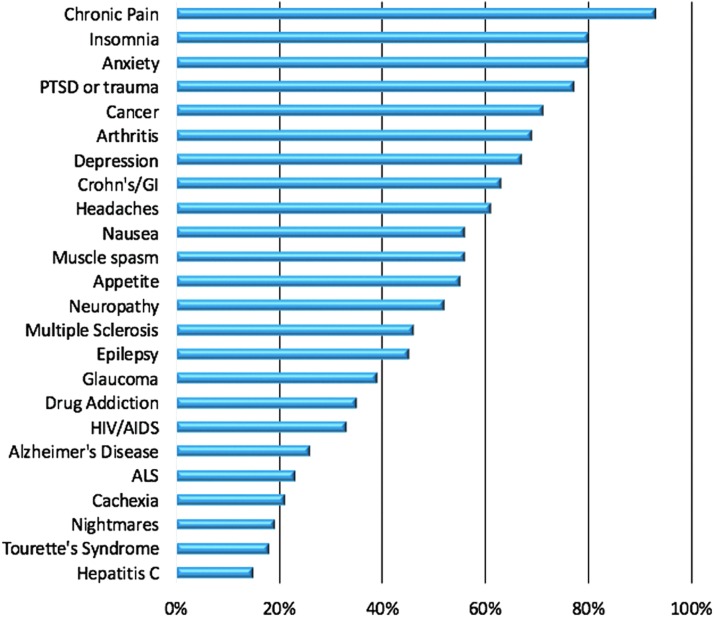
The percentage of dispensary staff who reported frequently seeing patients with the following symptoms or conditions. ALS, amyotrophic lateral sclerosis; GI, gastrointestinal; PTSD, post-traumatic stress disorder.

Dispensary staff members were queried on the symptoms or conditions frequently reported by their patients (see Fig. 1 for a summary). The most frequent symptoms included chronic pain (93%; *n*=41), insomnia (80%; *n*=35), and anxiety (80%; *n*=35). Approximately two-thirds of the sample (62%; *n*=26) reported that they always or often check in or follow-up about their patients' health status.

### Dispensary staff recommendations

Dispensary staff were queried regarding the specific recommendations they make to patients and on what information those recommendations are based. A majority (94%; *n*=47) reported that they provide advice, guidance, or counsel to patients. The type of advice included information on particular cannabis strains (88%; *n*=44), suggested administration methods (88%; *n*=44), potential cannabis side effects (80%; *n*=40), benefits of cannabis for specific symptoms (74%; *n*=37), and other recommendations (22%; *n*=11) such as natural remedies, travel/shipping legal advice, dosing guidelines, and ailment or disease-specific information. Those who did not provide advice or counsel indicated that it was not part of their role at the dispensary (i.e., driver or delivery service). None of the dispensary staff members indicated that they provide advice regarding medications or drugs other than cannabis to their patients.

Dispensary staff reported making recommendations for specific strains or formulas of cannabis based on the following: the particular condition or ailment (89%; *n*=39), the experience of other patients (83%; *n*=35), patient preference or needs (79%; *n*=33), their own experience (71%; *n*=30), information obtained from scientific articles (68%; *n*=28), dispensary owner or other staff recommendations (52%; *n*=22), information obtained on websites (48%; *n*=20), new variety or unusual/rare breed (47%; *n*=20), and what needs to get moved out of inventory (21%; *n*=9).

The survey assessed which plant strains (i.e., sativa, indica, hybrid) and which cannabinoid concentrations (i.e., high THC, high CBD, 1:1 ratio of THC/CBD) were recommended for particular symptoms or conditions. Several patient conditions were associated with specific plant strain recommendations by dispensary staff (Table 1). Indeed, dispensary staff were more likely to recommend indica for chronic pain and Tourette's syndrome than sativa. They were also more likely to recommend indica for insomnia, anxiety, and nightmares than both sativa and hybrid plants. They were more likely to recommend indica and hybrid plants for PTSD or trauma, and muscle spasms compared to sativa. In contrast, staff were less likely to recommend indica for depression than sativa or hybrid plants. Finally, dispensary staff recommended hybrid strains more often for ALS than sativa.

**Table 1.** **Dispensary Staff Plant Strain Recommendations for Patient Symptoms or Conditions**^a^

Symptom or condition	S, %	I, %	H, %	Significance
ALS or Lou Gehrig's disease	11	21	36	*t*(27)=2.07, *p*=0.048 (S vs. H)
Alzheimer's disease	14	18	18	ns
Anxiety	13	60	23	*t*(29)=3.61, *p*=0.001 (S vs. I)
				*t*(29)=2.43, *p*=0.021 (I vs. H)
Appetite	53	33	33	ns
Arthritis	25	36	32	ns
Cachexia (wasting syndrome)	32	36	32	ns
Cancer	36	36	46	ns
Chronic pain	23	57	30	*t*(29)=2.25, *p*=0.032 (S vs. I)
Crohn's disease/GI	21	43	25	ns
Depression	63	16	47	*t*(29)=3.41, *p*=0.002 (S vs. I)
				*t*(29)=2.32, *p*=0.027 (I vs. H)
Drug addiction	27	36	39	ns
Epilepsy	14	18	18	ns
Glaucoma	32	25	32	ns
Headaches or migraines	27	23	37	ns
Hepatitis C	18	21	25	ns
HIV/AIDS	27	36	32	ns
Insomnia	3	80	20	*t*(29)=8.66, *p*=0.000 (S vs. I)
				*t*(29)=4.11, *p*=0.000 (I vs. H)
Multiple sclerosis	18	29	36	ns
Muscle spasms	7	40	33	*t*(29)=3.01, *p*=0.005 (S vs. I)
				*t*(29)=2.47, *p*=0.020 (S vs. H)
Nausea	27	33	47	ns
Neuropathy	17	43	40	ns
Nightmares	13	70	20	*t*(29)=4.39, *p*=0.000 (S vs. I)
				*t*(29)=3.40, *p*=0.002 (I vs. H)
PTSD or trauma	11	46	36	*t*(27)=2.77, *p*=0.010 (S vs. I)
				*t*(27)=2.07, *p*=0.058 (S vs. H)
Tourette's syndrome	11	39	25	*t*(27)=2.28, *p*=0.031 (S vs. I)

^a^ Percentages do not add up to 100% because participants selected each symptom/condition for which they recommended a given strain, independently. Thus, symptoms/conditions for which percentages added up to above 100% indicate that some participants recommended multiple strains for a given condition. Conversely, symptoms/conditions for which percentages added up to less than 100% indicate that some participants did not recommend any strain for a given condition.

GI, gastrointestinal; H, hybrid; I, indica; ns, not significant; PTSD, post-traumatic stress disorder; S, sativa.

In terms of specific cannabinoid recommendations, dispensary staff were more likely to recommend a 1:1 ratio of THC:CBD for anxiety, PTSD or trauma, and Crohn's disease compared to high THC. They were more likely to recommend high CBD and a 1:1 ratio for ALS, epilepsy, and muscle spasms compared to high THC. Dispensary staff were more likely to recommend high CBD than high THC for arthritis and Alzheimer's disease. They were also more likely to recommend a 1:1 ratio for hepatitis C compared to high THC or high CBD. Staff were more likely to recommend high THC for appetite than high CBD (Table 2).

**Table 2.** **Dispensary Staff Cannabinoid Recommendations for Patient Symptoms or Conditions**^a^

Symptom or condition	High THC (THC), %	High CBD (CBD), %	1:1 Ratio THC/CBD (1:1), %	Significance
ALS or Lou Gehrig's disease	18	57	57	*t*(27)=2.67, *p*=0.013 (THC vs. CBD, THC vs. 1:1)
Alzheimer's disease	21	61	50	*t*(27)=2.61, *p*=0.015 (THC vs. CBD)
Anxiety	13	30	40	*t*(29)=2.19, *p*=0.037 (THC vs. 1:1)
Appetite	63	10	7	*t*(29)=4.93, *p*=0.000 (THC vs. 1:1)
				*t*(29)=4.33, *p*=0.000 (THC vs. CBD)
Arthritis	32	71	53	*t*(27)=2.20, *p*=0.036 (THC vs. CBD)
Cachexia (wasting syndrome)	54	32	43	ns
Cancer	50	57	68	ns
Chronic pain	33	53	57	ns
Crohn's disease/GI	25	47	61	*t*(27)=2.23, *p*=0.034 (THC vs. 1:1)
Depression	33	26	53	ns
Drug addiction	36	42	50	ns
Epilepsy	7	61	54	*t*(27)=4.59, *p*=0.000 (THC vs. CBD)
				*t*(27)=3.99, *p*=0.000 (THC vs. 1:1)
Glaucoma	39	36	50	ns
Headaches or migraines	33	37	63	ns
Hepatitis C	18	29	64	*t*(27)=3.12, *p*=0.004 (THC vs. 1:1)
				*t*(27)=2.07, *p*=0.049 (CBD vs. 1:1)
HIV/AIDS	36	36	68	ns
Insomnia	33	33	40	ns
Multiple sclerosis	32	46	57	ns
Muscle spasms	10	53	53	*t*(29)=3.53, *p*=0.001 (THC vs. CBD, THC vs. 1:1)
Nausea	50	23	53	ns
Neuropathy	20	47	47	ns
Nightmares	20	43	37	ns
PTSD or trauma	21	28	57	*t*(27)=2.36, *p*=0.026 (THC vs. 1:1)
Tourette's syndrome	18	43	43	ns

^a^ Percentages do not add up to 100% because participants selected each symptom/condition for which they recommended a given cannabinoid concentration, independently. Thus, symptoms/conditions for which percentages added up to above 100% indicate that some participants recommended multiple cannabinoid concentrations for a given condition. Conversely, symptoms/conditions for which percentages added up to less than 100% indicate that some participants did not recommend any cannabinoid concentration for a given condition.

CBD, cannabidiol; THC, tetrahydrocannabinol.

### Dispensary staff attitudes

Dispensary staff members were asked to rate satisfaction with their current position on a scale ranging from very dissatisfied (1) to very satisfied (5). The mean score for satisfaction was 4.27 (SD=0.95, *n*=41), indicating a high level of satisfaction with their work.

Other attitudes toward dispensary work were assessed on a scale ranging from never (1) to always (5). Items included the following: feeling valued or appreciated because of work (M=3.81, SD=1.13, *n*=42), feeling stigmatized or looked down upon by others for their work (M=2.57; SD=1.06, *n*=42), and experiencing burnout or fatigue as a result of work (M=2.83, SD=1.10, *n*=42).

## Discussion

While a number of studies have examined the characteristics of patients seeking cannabis recommendations from a physician,^3,8^ and those obtaining cannabis from a dispensary,^2,9^ the present study serves as the first examination of characteristics and practices of dispensary staff. Findings indicate that the vast majority of staff provide specific counseling to patients regarding cannabis that may be most helpful for their individual conditions, and that these recommendations are based on a number of sources, both empirically and nonempirically based. Despite the vast number of staff members offering recommendations to patients, only 20% of our sample reported prior medical and/or scientific training.

In terms of plant strain recommendations, very little work has documented patient preference or the specific efficacy of certain cannabis strains as a function of clinical condition. Staff recommendation of indica strains for chronic pain in the current study was consistent with individual patient preference observed in other work.^10^ The recommendation of indica primarily for insomnia and nightmares is consistent with one patient survey,^11^ but differs from another empirical study that highlighted patient preference for sativa strains for sleep difficulties.^12^ While observations related to strain recommendations are interesting, due to extensive hybridization and variations in growing conditions, the differences between cannabis strains do not seem to play as large a role in determining subjective effects as cannabinoid concentrations.^13^ This has led some to argue that distinctions among cannabis chemovars labeled as “Sativa” or “Indica” are relatively meaningless unless accompanied by detailed accurate assays of cannabinoid and terpenoid content.^14^

Dispensary staff recommendations of cannabinoid concentrations (e.g., THC, CBD) for particular patient symptoms and conditions were also frequently incongruent with the existing empirical literature. For instance, the existing empirical literature would suggest that cannabis high in CBD could be particularly helpful for individuals with anxiety disorders, while cannabis high in THC could actually *lead to* acute and long-term anxiety reactions.^6,15,16^ Early pre-clinical work has also highlighted CBD as a potential antidepressant,^15^ anticonvulsant,^16,17^ and therapeutic for PTSD.^15^ On the other end of the spectrum, the literature would suggest that THC may be an antiemetic, and particularly beneficial for appetite stimulation and pain.^18^ Finally, a number of reviews have documented that a combination of THC and CBD (e.g., nabiximols) can have therapeutic value for individuals with spasticity due to multiple sclerosis,^16,19^ neuropathic pain,^16,20^ as well as sleep disturbances.^15^ Based on the above literature, it appears that a meaningful number of dispensary staff are providing recommendations for cannabinoids that have either not been shown to be effective for a given condition (e.g., 33% recommending THC for depression, 10% recommending CBD for appetite, 78% recommending either high THC or high CBD for multiple sclerosis), or could actually worsen a patient's condition (e.g., 13% recommending THC for anxiety, 7% recommending THC for epilepsy).

Aside from the present study's contribution to the literature in terms of describing the characteristics of cannabis dispensary staff, findings highlight the importance of consistent, evidence-based training of those providing specific recommendations of cannabis strains or cannabinoid concentrations for a given patient condition (e.g., physicians, dispensary staff). Indeed, while one might expect the most qualified individuals to provide specific recommendations of cannabis product to be trained physicians who are aware of a patient's medical history and other prescription medications that could interact with certain cannabinoids,^21–23^ it is dispensary staff who are the most likely to provide cannabis advice. As each state is currently responsible for drafting and monitoring its own cannabis legislation, it is imperative for states to mandate some form of educational certification for *any* individual providing cannabis advice to patients, not just physicians. Furthermore, given the quickly evolving literature in this field, it seems necessary for individuals to receive regular updates *via* continuing medical education. Given the increasing availability and preferences for myriad methods of cannabis consumption (e.g., edibles, extracts, dabs)^2,24^ and corresponding risks,^25,26^ patient and provider education programs should strive to include trainings in safe and effective use of cannabis as a function of preparation.

The present study data, although novel, are not without limitations. First, given the cross-sectional nature of the investigation, we were unable to determine changes in staff behavior over time or as a function of the implementation of particular training programs. Second, while the utilization of structured surveys allowed for the systematic and quantitative analysis of staff characteristics and practices, such surveys are limited in that they do not allow for nuanced information regarding staff behavior. Future prospective qualitative work that incorporates open-ended questions would aid in fleshing out the present findings. Third, although both medicinal and nonmedical dispensary staff were recruited, higher response rates in specific states may limit generalizability of findings to dispensaries across the United States or in other countries. Web-based recruitment methods also limit our ability to determine overall sample representativeness. Indeed, the survey response rate may have been restricted by perceived stigma associated with both medical and nonmedical cannabis use and/or skepticism of researchers by dispensary staff. Finally, the current study aimed to describe overall behavior among dispensary staff, and in doing so the survey did not collect information about physician practices or detailed information about the provision of services for certain, particularly vulnerable, populations (e.g., individuals with PTSD). Limitations notwithstanding, the present study serves as a “call to action” for consistent and evidence-based training of providers in the cannabis industry.

## Abbreviations Used

ALS: amyotrophic lateral sclerosis
CBD: cannabidiol
GI: gastrointestinal
METRC: Marijuana Enforcement Tracking Reporting Compliance
PTSD: post-traumatic stress disorder
THC: tetrahydrocannabinol
